# Climatic Associations of Akabane Virus Occurrence in East Asia: Temperature-Driven Patterns Based on the Köppen–Geiger Classification

**DOI:** 10.3390/microorganisms14081837

**Published:** 2026-08-19

**Authors:** Jung Won Kim, Jung-Yong Yeh

**Affiliations:** 1 Department of Life Sciences, College of Life Sciences and Bioengineering, Incheon National University, Bio-Complex, Harmorny-ro 265, Yeonsu-gu, Incheon 22014, Republic of Korea; 2 Center for Brain-Machine Interface, Incheon National University, Bio-Complex, Harmorny-ro 265, Yeonsu-gu, Incheon 22014, Republic of Korea

**Keywords:** Akabane virus, *Culicoides*, Köppen–Geiger climate classification

## Abstract

Akabane virus (AKAV) is a *Culicoides*-borne arbovirus that causes congenital malformations and reproductive losses in ruminants, resulting in substantial economic losses in livestock production. Because vector activity and virus transmission are strongly influenced by environmental conditions, defining climatic factors associated with AKAV distribution is critical for understanding its epidemiology. However, such relationships have not been systematically evaluated in East Asia. In this study, we applied the Köppen–Geiger climate classification to characterize regional climatic zones in South Korea and Japan and examined their associations with AKAV case counts. AKAV cases were predominantly observed in temperate climate zones (Cfa and Cwa). Temperature-related variables showed consistent positive associations with AKAV case counts, with a 1 °C increase in annual mean temperature associated with approximately 1.38–1.56-fold increases in reported cases. In contrast, precipitation variables exhibited weak or negative associations. These findings indicate that temperature is an important climatic factor associated with AKAV case counts, suggesting that climate-dependent modulation of vector dynamics may contribute to observed patterns of AKAV occurrence. This study provides a climate-based framework for understanding the spatial distribution of AKAV and supports the development of targeted surveillance and control strategies under changing environmental conditions.

## 1. Introduction

Akabane virus (AKAV) is a member of the family Peribunyaviridae, genus Orthobunyavirus, and belongs to the Simbu serogroup, which includes Aino virus, Schmallenberg virus, and Peaton virus [1]. These viruses share antigenic and genetic characteristics and are recognized as important pathogens of ruminants [2]. AKAV infection is associated with severe reproductive disorders, including abortion, stillbirth, and congenital malformations such as arthrogryposis–hydranencephaly syndrome [3]. In addition to reproductive impacts, neurological manifestations have occasionally been reported in infected animals [4]. Because AKAV is transmitted among cattle, sheep, and goats, outbreaks can spread rapidly within livestock populations, resulting in substantial economic losses due to decreased productivity and increased control costs [5].

AKAV is widely distributed across temperate and tropical regions, including Southeast Asia, the Middle East, Australia, and Africa [6]. This broad distribution reflects both the ecological adaptability of the virus and the widespread presence of its primary vectors, *Culicoides* biting midges [7,8,9,10]. East Asia is considered a high-risk region for AKAV outbreaks, where epizootics often coincide with seasonal increases in vector activity [11,12]. Historical outbreaks illustrate the magnitude of impact; for example, the 1972–1973 epidemic in Japan resulted in approximately 31,000 cases of reproductive failure and congenital abnormalities [13,14,15]. Subsequent outbreaks have been periodically reported in Japan, South Korea, and neighboring countries, frequently during warmer seasons [12,16,17]. Serological surveys further suggest that AKAV may circulate subclinically in endemic regions, even in the absence of reported outbreaks [16,18,19,20].

The transmission dynamics of AKAV are closely linked to environmental conditions, particularly temperature, which strongly influences the activity and distribution of *Culicoides* vectors [21,22]. Despite increasing recognition of the role of climate in shaping arbovirus epidemiology, most previous studies have focused on future projections or historical trends, with limited emphasis on present-day spatial patterns of viral occurrence. In particular, a systematic and quantitative assessment of the relationship between climatic conditions and AKAV distribution in East Asia remains limited.

In this study, we investigated the geographical distribution of AKAV and evaluated its association with climatic factors in South Korea and Japan using the Köppen–Geiger climate classification and meteorological data. Unlike analyses based solely on individual meteorological variables, the Köppen–Geiger climate classification integrates long-term temperature and precipitation characteristics into ecologically relevant climate zones. This framework facilitates a more comprehensive evaluation of broad-scale climatic patterns associated with AKAV occurrence across different geographic regions. By integrating epidemiological and environmental datasets, this study aims to identify climatic factors associated with AKAV case counts, particularly temperature-driven patterns, and to provide a framework for understanding climate-associated patterns relevant to surveillance in endemic regions.

## 2. Materials and Methods

### 2.1. Climate Data

**Figure 1:**
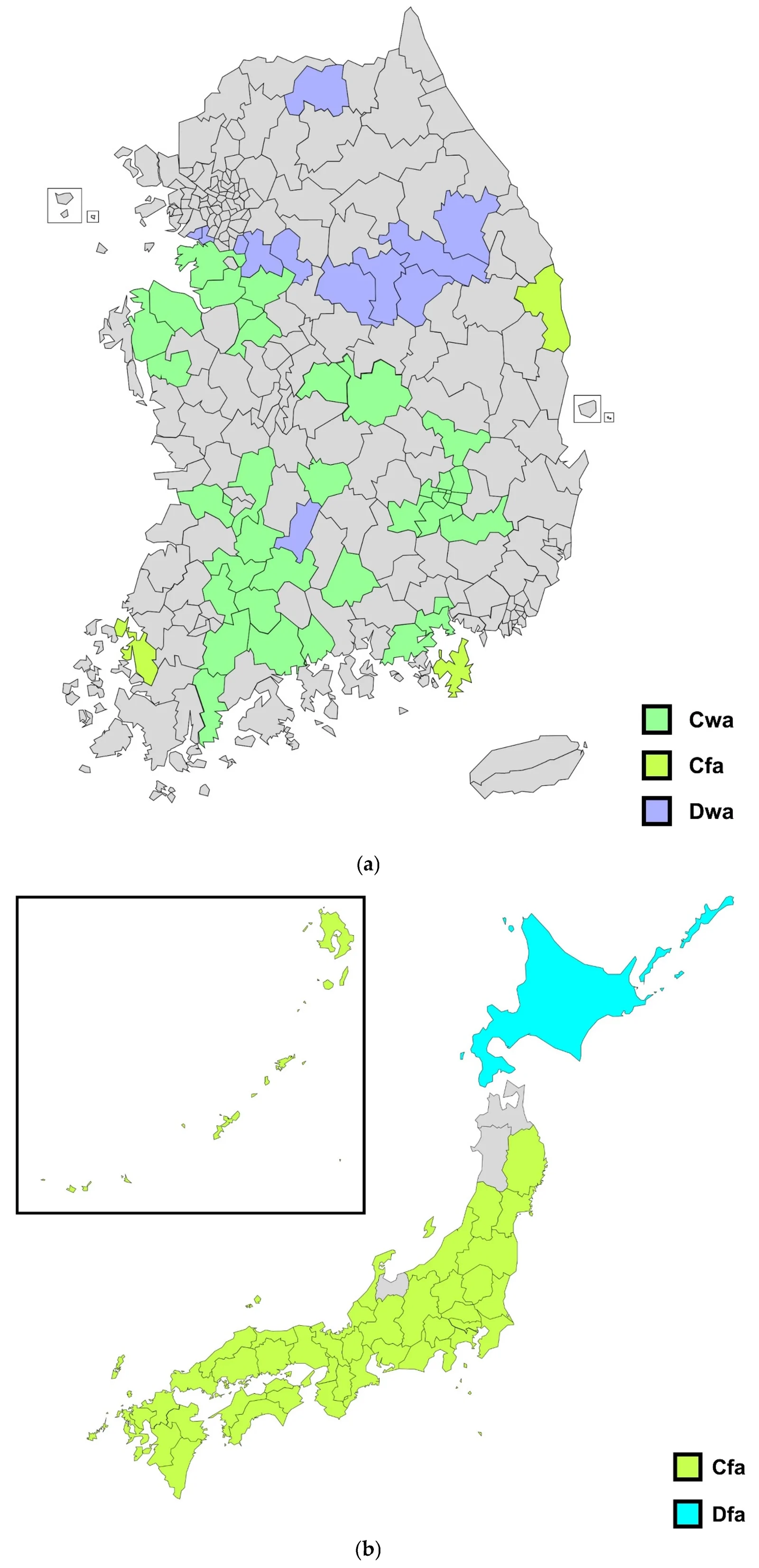
Köppen–Geiger (KG) climate classification of South Korea and Japan regions where Akabane virus (AKAV) outbreaks have been previously reported. In South Korea, temperate and warm climates are distributed in southern and coastal regions, whereas colder climates predominate inland (**a**). In Japan, humid temperate climates are predominant, with colder zones restricted to northern regions (**b**). The inset box represents the southern island regions, including Kagoshima and Okinawa prefectures. Climate zones include Cwa (temperate, dry winter, hot summer), Cfa (temperate, no dry season, hot summer), Dwa (cold, dry winter, hot summer), and Dfa (cold, no dry season, hot summer).

The Köppen–Geiger (KG) climate classification is one of the most widely used frameworks for classifying global climates [23]. It categorizes climates into five major groups (tropical, arid, temperate, continental, and polar), which are further subdivided based on temperature and precipitation patterns [24,25]. Based on meteorological data from regions in South Korea and Japan where AKAV cases were previously reported, the climatic parameters required for KG classification were calculated. Because the objective of this study was to characterize climatic associations among affected regions rather than to predict disease occurrence across all geographic areas, locations without reported AKAV cases were excluded from the analysis. Therefore, this study evaluated climatic characteristics within regions with reported AKAV case counts rather than estimating absolute risk across all geographic areas. Using these parameters, KG zones were assigned to 85 regions (41 in South Korea and 44 in Japan) using a standard KG classification approach implemented in R (version 4.5.1) [26]. The resulting classifications included Cwa, Cfa, and Dwa in South Korea and Cfa and Dfa in Japan (Figure 1). Detailed descriptions of KG classification criteria are provided in Supplementary Table S1–S3 [25,27].

### 2.2. Meteorological Data

Thirty years (1991–2020) of meteorological data for South Korea and Japan were obtained from the respective national meteorological agencies: the Korea Meteorological Administration and the Japan Meteorological Agency. These data were used to derive long-term climatological averages for each region. AKAV case data were spatially matched to the corresponding regional meteorological data based on administrative units. Monthly mean temperature and precipitation data were aggregated to derive the climatic parameters, which were subsequently used for the KG climate classification.

### 2.3. AKAV Occurrence and Case Data in South Korea and Japan

Data on AKAV cases in South Korea from 2000 to 2020 were obtained from the Animal and Plant Quarantine Agency. These data were reported at the municipal-level administrative units (si, gun, and gu). In contrast, data for Japan were available at the prefectural level. Detailed AKAV case numbers for Japan between 1998 and 2020 were obtained from the Annual Report on Surveillance of Infectious Diseases issued by the Ministry of Agriculture, Forestry and Fisheries of Japan. Because spatial resolution differed between countries, analyses were conducted using the finest available administrative units for each dataset, and results were interpreted with caution due to differences in geographic scale. Data prior to 2000 for South Korea and prior to 1998 for Japan were not available; therefore, the analysis was restricted to the available surveillance period, and the results reflect patterns within this timeframe.

### 2.4. Cattle Population

Cattle population data for South Korea and Japan were obtained from official governmental sources, including the Korea Institute for Animal Products Quality Evaluation (KAPE) and the Portal Site of Official Statistics of Japan (e-STAT). Because consistent long-term population data were not available for the entire study period, cattle population data from 2016 to 2020 were used to represent regional population sizes. For each administrative unit, the mean cattle population over this period was calculated and matched to the corresponding AKAV case counts by administrative unit. These values were used as an offset variable in the regression analysis to account for differences in population size across regions. Detailed summaries of meteorological data, AKAV case counts, and cattle population are provided in Supplementary Tables S4 (South Korea) and S5 (Japan).

### 2.5. Data Integration of South Korea and Japan

For South Korea, AKAV case counts and meteorological data were organized at the municipal level (si, gun, and gu), whereas in Japan, the prefecture was used as the administrative unit. Datasets from the two countries were integrated based on their geographic proximity and broadly comparable climatic and ecological characteristics. Both South Korea and Japan are located within the mid-latitude zone (approximately 30–45 °N) in East Asia and share similar temperate climate conditions and seasonal patterns [28]. In addition, the distribution and activity patterns of *Culicoides* vectors are broadly comparable between the two countries [29,30,31]. Because spatial resolution differed between datasets, analyses were conducted using the finest available administrative units for each country, and results were interpreted with caution due to differences in geographic scale.

### 2.6. Statistical Analysis

The relationship between AKAV case counts and KG climate zones was evaluated using the Wilcoxon rank-sum test. Regions were classified into KG zones based on their climatic characteristics (South Korea at the municipal level and Japan at the prefectural level) and subsequently grouped into warm (C) and cold (D) climate categories. Differences in AKAV case counts between these groups were assessed. To further evaluate the relationship between climatic variables and AKAV case counts, negative binomial regression analysis was performed using regional case counts as the dependent variable. Temperature and precipitation variables, which are key components of the KG classification and major factors influencing *Culicoides* vector activity, were included as independent variables. To minimize multicollinearity, each model included only one temperature-related variable and one precipitation-related variable. The negative binomial regression model was specified as:

$$\log\left(\mu \right)=\log\left(A\right)+\;\beta 0\;+\;\beta 1X1\;+\;\beta 2X2\;+\;\ldots \;+\;\beta pXp$$

where μ is the expected number of cases, X_1_–X_p_ are independent variables, β_0_–β_p_ are regression coefficients, and A represents the cattle population used as an offset variable to account for regional differences in population at risk. All statistical analyses were performed using R software (ver 4.5.1) with the VGAM package [26], and statistical significance was defined as *p* < 0.05. Model diagnostics included assessment of overdispersion and multicollinearity using the dispersion statistic and variance inflation factor (VIF), respectively. Model fit was further evaluated by comparing Akaike Information Criterion (AIC) values between the negative binomial models and the corresponding Poisson models. Because regions without reported AKAV cases were excluded, the analysis reflects associations within affected regions rather than population-level risk.

The relationship between annual AKAV case counts and meteorological variables (annual mean temperature and annual total precipitation, Supplementary Table S6) was further evaluated using Spearman’s rank correlation analysis in Okayama Prefecture, Japan. Okayama Prefecture was selected because it had the highest cumulative number of reported AKAV cases during the study period (1998–2020) and provided continuous annual surveillance data, allowing temporal comparison with long-term meteorological records. To assess potential delayed associations, both same-year and previous-year meteorological variables were examined.

## 3. Results

### 3.1. Distribution of AKAV in South Korea and Japan

**Figure 2:**
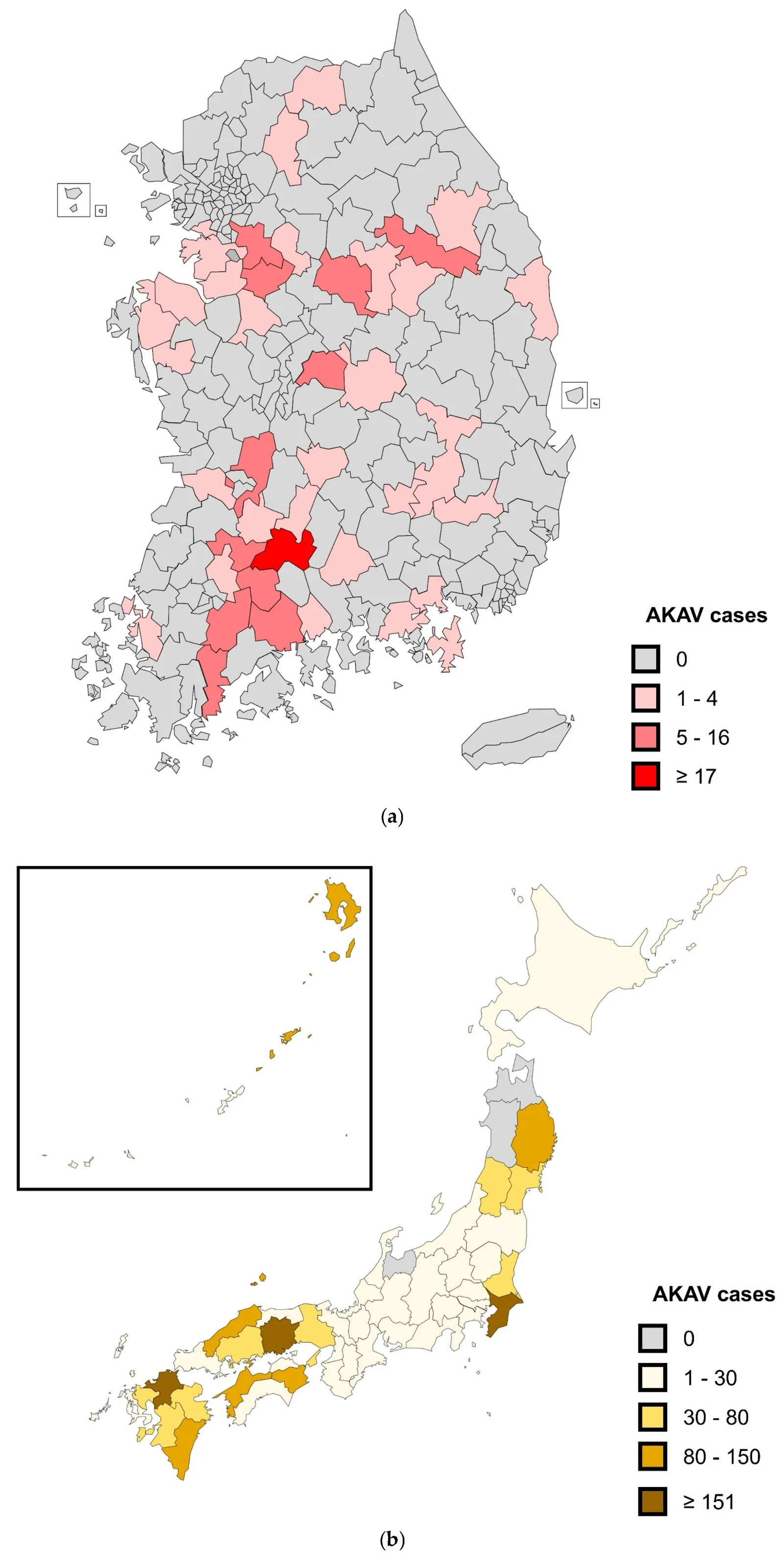
Spatial distribution of Akabane virus (AKAV) cases in South Korea and Japan. Case intensity increases with darker color shading: red scale for South Korea (**a**) and yellow scale for Japan (**b**). The inset box represents the southern island regions, including Kagoshima and Okinawa prefectures.

The spatial distribution of regions with reported AKAV cases in South Korea (2000–2020) and Japan (1998–2020) is shown in Figure 2. Although AKAV was reported across both countries, case counts were unevenly distributed. In South Korea, higher case counts were observed in the southwestern regions, particularly in Jeollabuk-do and Jeollanam-do (Supplementary Table S4). Most cases (178) were recorded in temperate climate zones, including Cwa (175) and Cfa (3), with fewer cases in Dwa (42) (Table 1). In Japan, AKAV was detected in most prefectures, with relatively higher case counts in southwestern and central regions, including Okayama and Chiba (Supplementary Table S5). Within the KG classification, the majority of reported cases occurred in the Cfa climate zone (2091), with substantially fewer cases in Dfa (20) (Table 1).

Overall, AKAV cases in both countries were primarily observed in temperate climate zones. When combined, the highest number of cases was observed in the Cfa zone (2094), followed by Cwa (175), Dwa (42), and Dfa (20). These results show that reported AKAV cases were predominantly distributed in temperate climate zones, particularly Cfa and Cwa. Cfa and Cwa are temperate climate zones characterized by hot summers, whereas Dfa and Dwa represent colder continental climates; additionally, Cwa and Dwa are characterized by dry winters, whereas Cfa and Dfa have no dry season.

### 3.2. Wilcoxon Rank-Sum Test

The Wilcoxon rank-sum test indicated a significant difference in AKAV case counts between climate groups (W = 640.5, *p* < 0.05). Regions classified as temperate climates (Cwa and Cfa) had higher case counts than regions in colder climate zones (Dwa and Dfa) (Table 2).

### 3.3. Negative Binomial Regression

Negative binomial regression analysis showed that temperature-related variables showed consistent positive associations with AKAV case counts, whereas precipitation variables showed weaker or inverse associations (Table 3). Among the temperature parameters, annual mean temperature (Ta) showed a significant positive association with AKAV case counts across all models (*p* < 0.05). The regression coefficients for Ta ranged from 0.3237 to 0.4429, corresponding to incidence rate ratios (IRRs) of 1.38 to 1.56. This indicates that a 1 °C increase in Ta was associated with approximately 1.38–1.56-fold increases in AKAV case counts, depending on the precipitation variable included in the model.

Similarly, the monthly mean temperature of the warmest month (Tmax) and the coldest month (Tmin) were also significantly associated with AKAV case counts (*p* < 0.05). Tmax showed a stronger association (*β* = 0.5777; IRR ≈ 1.78) compared with Tmin (*β* = 0.2926; IRR ≈ 1.34). In contrast, several precipitation variables, including annual mean precipitation (Pa), maximum precipitation in the summer half-year (Psmax), and minimum precipitation in the summer half-year (Psmin), showed weak negative associations with AKAV case counts. The estimated coefficients were small (e.g., Pa: *β* ≈ −0.0014; IRR ≈ 0.9986), suggesting limited effect sizes. Other precipitation variables (Pmin, Pwmax, and Pwmin) were not statistically significant (*p* ≥ 0.05). Model diagnostics indicated substantial overdispersion (dispersion = 51.19–85.92), supporting the use of negative binomial regression. Variance inflation factor (VIF) values were below 5 for all variables, indicating no evidence of multicollinearity [32,33]. The negative binomial models consistently showed substantially lower AIC values (776.74–793.91) than the corresponding Poisson models (3285.52–4340.41), indicating better relative model fit [34].

### 3.4. Association Between AKAV Cases and Meteorological Data in Okayama Prefecture

**Figure 3:**
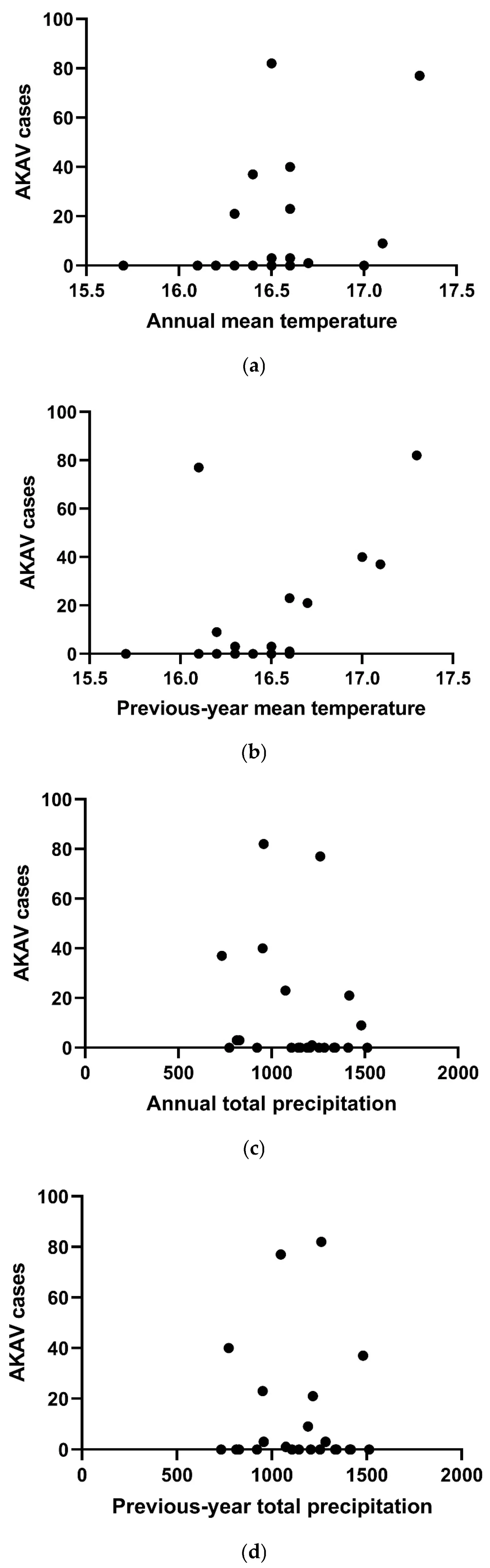
Scatter plots of Spearman’s rank correlation showing the relationship between annual meteorological variables (annual mean temperature and total precipitation) and Akabane virus (AKAV) case counts in Okayama Prefecture, Japan (1998–2020). (**a**) Same-year temperature (1998–2020). (**b**) Previous-year temperature (1997–2019). (**c**) Same-year precipitation (1998–2020). (**d**) Previous-year precipitation (1997–2019).

Spearman’s rank correlation analysis showed significant correlations between temperature variables and AKAV case counts in Okayama Prefecture. AKAV case counts from 1998 to 2020 were positively correlated with annual mean temperature (*p* < 0.05, rs = 0.51). A similar positive correlation was observed between AKAV case counts and the annual mean temperature of the preceding year (*p* < 0.05, rs = 0.48). In contrast, no significant correlation was observed between AKAV case counts and annual total precipitation (*p* ≥ 0.05) (Figure 3).

## 4. Discussion

This study showed that AKAV case counts in South Korea (2000–2020) and Japan (1998–2020) were unevenly distributed across KG climate zones. Most cases were observed in temperate regions (Cwa and Cfa), whereas fewer cases were reported in colder zones (Dwa and Dfa). The Köppen–Geiger classification reflects long-term temperature and precipitation patterns rather than temperature alone. The predominance of AKAV cases in the Cfa and Cwa climate zones suggests that the broader climatic characteristics of temperate regions may also be associated with the spatial distribution of AKAV cases.

However, negative binomial regression analysis further indicated that temperature-related variables showed the most consistent association with regional AKAV case counts. These findings suggest that temperature is an important climatic factor associated with the geographic distribution of AKAV. However, this study was designed to characterize climatic patterns among regions with reported AKAV case counts and does not estimate absolute risk across all geographic regions. In contrast to temperature, several precipitation variables showed weak or inverse associations with AKAV case counts. Although intense rainfall and high humidity may influence vector habitats, these effects were not directly assessed in this study. The observed patterns are consistent with previous reports suggesting that environmental conditions can influence *Culicoides* habitat stability and vector survival [35,36,37]. Some precipitation variables did not show statistically significant associations. This may reflect differences in the ecological characteristics of *Culicoides* compared with other vector insects. Unlike mosquitoes, which depend on standing water for larval development, *Culicoides* species utilize a variety of moist substrates, including soil and organic matter, which may reduce their sensitivity to precipitation variability [38,39].

In Okayama Prefecture, temperature showed a significant correlation with AKAV case counts in the Spearman correlation analysis. Years with relatively higher temperatures tended to coincide with increased AKAV incidence. These findings provide regional evidence consistent with the nationwide associations observed in the negative binomial regression analysis, suggesting that temporal variation in AKAV case counts may reflect broader temperature-related patterns. However, because this analysis was conducted in a single high-incidence region, the findings should be interpreted as region-specific observations and may not be directly generalizable.

Previous studies have reported that arbovirus epidemiology is influenced by climatic conditions affecting vector dynamics [21]. The present study extends these observations by comparing patterns across two East Asian countries, showing that temperature-related variables were consistently associated with AKAV case counts. A 1 °C increase in annual mean temperature was associated with approximately 1.38–1.56-fold increases in reported cases, with similar trends observed for Tmax and Tmin.

These findings are relevant in the context of ongoing climate change. Global surface temperatures have increased by approximately 1.28 °C relative to the 20th-century baseline [40,41] and long-term warming trends have also been observed in South Korea [42]. Such changes may influence the distribution and activity of *Culicoides* vectors, potentially affecting AKAV occurrence patterns. Future climate projections suggest that temperate climate zones may expand in East Asia, which could alter the spatial distribution of AKAV risk [43,44]. These changes may extend the seasonal window of vector activity and highlight the importance of climate-informed surveillance strategies.

Several limitations should be acknowledged. First, spatial resolution differed between countries, with municipal-level data in South Korea and prefectural-level data in Japan. This difference reflects the finest administrative resolution available from the official surveillance data sources in each country. The coarser spatial resolution of the Japanese data may obscure within-prefecture variation in AKAV case counts and climatic conditions, potentially limiting direct comparability with the finer municipal-level data available for South Korea. Therefore, differences in spatial resolution may affect direct comparability between South Korea and Japan and should be considered when interpreting regional-level associations. Second, historical case data for South Korea prior to 2000 and Japan prior to 1998 were not available. Third, this study focused on climatic factors and did not incorporate other ecological determinants that may influence AKAV occurrence, including *Culicoides* distribution and abundance, livestock density, vaccination programs, animal movement, differences in surveillance intensity, and diagnostic practices between regions. In particular, differences in surveillance intensity and diagnostic practices between countries and over time may also have influenced the number and spatial distribution of reported AKAV cases. These factors were beyond the scope of the present study and should be considered in future investigations. Fourth, because regions without reported AKAV cases were excluded from the analysis, the regression results should be interpreted as climatic associations among affected regions rather than predictors of disease occurrence across the entire study area. Future studies incorporating both affected and unaffected regions could provide a more comprehensive assessment of climatic determinants of AKAV occurrence. Fifth, several limitations related to the regression analysis should be considered. Mean cattle population data from 2016–2020 were used as an offset for cumulative AKAV case counts covering the longer surveillance periods. Changes in regional cattle populations over these periods may therefore have affected the estimated incidence-rate relationships. In addition, residual diagnostics and sensitivity analyses beyond the reported assessments of overdispersion, multicollinearity, and model fit were not performed. Despite these limitations, consistent patterns observed across regions provide some support for the consistency of the observed associations. These findings also have implications for transboundary disease control. Because *Culicoides*-borne viruses can spread across regions, coordinated surveillance and monitoring efforts are required. Climate-driven changes in vector habitats may introduce new risks in previously unaffected areas, underscoring the importance of integrated approaches for managing AKAV and related arboviruses.

## Figures and Tables

**Table 1:** Distribution of Akabane virus (AKAV) case counts across Köppen–Geiger (KG) climate classification zones.

KG Classification	Climate	Number of Cases (South Korea)	Number of Cases (Japan)	Number of Cases (Total)
Cwa	Temperate	175	0	175
Cfa		3	2091	2094
Dwa	Cold	42	0	42
Dfa		0	20	20

Case counts are presented separately for South Korea and Japan, along with totals for each climate zone. Cases represent the number of confirmed AKAV cases in South Korea (2000–2020; si/gun/gu level) and Japan (1998–2020; prefectural level).

**Table 2:** The number of Akabane virus (AKAV) cases according to Köppen–Geiger (KG) climate classification in South Korea and Japan.

Group	KG Zones	AKAV Cases	Total
C	Cwa	175	2269
	Cfa	2094	
D	Dwa	42	62
	Dfa	20	
Total			2331

Regions were classified by KG climate zones based on AKAV case counts from South Korea (2000–2020) and Japan (1998–2020). Test statistic: W = 640.5, *p* < 0.05. Regions represent administrative units in South Korea (si/gun/gu) and Japan (prefectural level). Statistical significance was defined as *p* < 0.05.

**Table 3:** Results of negative binomial regression analysis of climatic factors associated with Akabane virus (AKAV) case counts in South Korea (2000–2020) and Japan (1998–2020).

Variables	*β* Coefficient	Standard Error	95% CI for β	IRR = e*^β^*	*p* Value
T_a_	0.4323	0.0556	(0.3261, 0.5449)	1.5408	*p* < 0.001 *
P_a_	−0.0014	0.0004	(−0.0022, −0.0006)	0.9986	*p* < 0.001 **
T_a_	0.3237	0.0381	(0.2490, 0.3984)	1.3823	*p* < 0.001 *
P_min_	−0.0026	0.0062	(−0.0148, 0.0096)	0.9974	*ns*
T_a_	0.3887	0.0460	(0.2985, 0.4789)	1.4751	*p* < 0.001 *
P_smax_	−0.0049	0.0012	(−0.0073, −0.0025)	0.9951	*p* < 0.001 **
T_a_	0.4429	0.0694	(0.3069, 0.5789)	1.5572	*p* < 0.001 *
P_smin_	−0.0143	0.0051	(−0.0243, −0.0043)	0.9858	*p* < 0.05 **
T_a_	0.3203	0.0406	(0.2407, 0.3999)	1.3775	*p* < 0.001 *
P_wmax_	−0.0004	0.0030	(−0.0063, 0.0055)	0.9996	*ns*
T_a_	0.3294	0.0346	(0.2616, 0.3972)	1.3902	*p* < 0.001 *
P_wmin_	−0.0069	0.0047	(−0.0161, 0.0023)	0.9931	*ns*
T_max_	0.5777	0.0681	(0.4442, 0.7112)	1.7819	*p* < 0.001 *
P_a_	−0.0011	0.0003	(−0.0017, −0.0005)	0.9989	*p* < 0.05 **
T_min_	0.2926	0.0422	(0.2099, 0.3753)	1.3399	*p* < 0.001 *
P_a_	−0.0013	0.0004	(−0.0021, −0.0005)	0.9987	*p* < 0.05 **

Cattle population (2016–2020) was included as an offset term in the model. *β*, regression coefficient; IRR, incidence rate ratio; CI, confidence interval. Statistical significance was set at *p* < 0.05. * indicates a statistically significant positive association, ** indicates a statistically significant negative association, and *ns* indicates a non-significant association.

## Data Availability

The original contributions presented in this study are included in the article/Supplementary Materials. Further inquiries can be directed to the corresponding author.

## Supplementary Materials

The following supporting information can be downloaded at: https://www.mdpi.com/article/10.3390/microorganisms14081837/s1, Table S1. Parameters used for Köppen–Geiger climate classification and their description. Table S2. Primary and secondary classification of Köppen–Geiger climate classification and its criteria. Table S3. Tertiary classification of Köppen–Geiger climate classification. Table S4. Regional Akabane virus cases and meteorological data in South Korea. Table S5. Regional Akabane virus cases and meteorological data in Japan. Table S6. Annual mean temperature and total precipitation data (1997–2020) and AKAV case numbers (1998–2020) of Okayama prefecture, Japan.
