# Supplementary material for: Climatic Associations of Akabane Virus Occurrence in East Asia: Temperature-Driven Patterns Based on the Köppen–Geiger Classification
Source: Microorganisms. 2026 Aug 19;14(8):1837. doi: 10.3390/microorganisms14081837 (PMC13515954; doi:10.3390/microorganisms14081837)
Supplement: Supplementary file 1 [file microorganisms-14-01837-s001.zip › microorganisms-4473248-supplementary.pdf]

Supplementary Tables

Table S1. Parameters used for Köppen–Geiger climate classification and their description.

Parameters	Description
T_a	Annual mean temperature (°C)
T_{max}	Monthly mean temperature of the warmest month (°C)
T_{min}	Monthly mean temperature of the coldest month (°C)
P_a	Annual total precipitation (mm)
P_{min}	Precipitation of the driest month (mm)
$P_{smax\ or\ smin}$	Extreme value of monthly precipitation for summer half-years (April to September, mm)
$P_{wmax\ or\ wmin}$	Extreme value of monthly precipitation for winter half-years (October to March, mm)
P_{th}	(i) if 70 % of P_a occurs in winter, then $P_{th} = 2 \times T_a$<br>(ii) if 70 % of P_a occurs in summer then $P_{th} = 2 \times T_a + 28$<br>(iii) when neither of the above two conditions then $P_{th} = 2 \times T_a + 14$

Table S2. Primary and secondary classification of Köppen–Geiger climate classification and its criteria. For the polar climates (E), only temperature-based criteria are applied, as no precipitation differentiation is defined. Therefore, the polar climates are classified first, followed by the arid (B), equatorial (A), and the temperate and snow climates (C and D), respectively.

Primary	Secondary	Description	Criteria
A ($T_{min} \geq 18$ ℃)	Af	Tropical rainforest, fully humid	$P_{min} \geq 60$ mm
	Am	Tropical monsoon	$P_a \geq 25$ (100 - Pmin)
	As	Equatorial savannah with a dry summer	$P_{min} < 60$ mm in summer
	Aw	Equatorial savannah with dry winter	$P_{min} < 60$ mm in winter
B ($P_a < 10\ P^{th}$)	BW	Desert	$P_a > 5$ Pth
	BS	Semi-arid or steppe	$P_a \leq 5$ Pth
C (-3 ℃ < T_{min} <18 ℃)	Cs	Warm temperate (or Mediterranean) with dry summer	$P_{smin} < P_{wmin}$, $P_{wmax} > 3\ P_{smin}$ and $P_{smin} < 40$mm
	Cw	Warm temperate (or Mediterranean) with dry winter	$P_{wmin} < P_{smin}$ and $P_{smax} > 10\ P_{wmin}$
	Cf	Warm temperate (or Mediterranean) fully humid	neither Cs nor Cw
D ($T_{min} \leq -3$ ℃)	Ds	Continental with dry summer	$P_{smin} < P_{wmin}$, $P_{wmax} > 3\ P_{smin}$ and $P_{smin} < 40$mm
	Dw	Continental with dry winter	$P_{wmin} < P_{smin}$ and $P_{smax} > 10\ P_{wmin}$
	Df	Continental, fully humid	neither Ds nor Dw
E ($T_{max} < 10$ ℃)	ET	Tundra	0 ℃ ≤ $T_{max} < +10$ ℃
	EF	Ice cap or frost	$T_{max} < 0$ ℃

Table S3. Tertiary classification of Köppen–Geiger climate classification.

Tertiary	Description	Criteria
h	Hot steppe or desert	$T_a \geq 18$ °C
k	Cold steppe or desert	$T_a < 18$ °C
a	Hot summer	$T_{max} \geq 22$ °C
b	Warm summer	Not a and at least 4 $T_{min} \geq 10$ °C
c	Cold summer and cold winter	Not b and $T_{min} > -38$ °C
d	Extremely continental	Like c but $T_{min} \leq -38$ °C

Table S4. Regional Akabane virus cases and meteorological data in South Korea.

Province	Region	KG zone	AKAV cases	T_a (℃)	T_{max} (℃)	T_{min} (℃)	P_a (mm)	P_{min} (mm)	P_{smax} (mm)	P_{smin} (mm)	P_{wmax} (mm)	P_{wmin} (mm)	P_{th}	Cattle population (head)
Gyeonggi	Ansan	Dwa	2	11.6	25.5	-3.3	1103.8	15.1	321.7	67.5	47.3	15.1	51.3	2,138
	Hwaseong	Cwa	1	12.3	25.8	-2.0	1102.2	13.5	327.3	63.3	46.0	13.5	52.7	52,780
	Pyeongtaek	Cwa	2	12.9	26.2	-2.0	1144.3	17.5	300.6	65.8	53.6	17.5	53.8	27,587
	Anseong	Cwa	6	12.5	26.1	-2.4	1194.9	19.0	323.1	68.5	50.7	19.0	53.0	101,723
	Gapyeong	Dwa	3	10.2	24.0	-5.3	1374.8	15.4	417.9	74.9	52.1	15.4	48.3	11,219
	Icheon	Dwa	4	11.7	25.0	-3.1	1315.9	19.2	364.3	78.5	50.9	19.2	51.4	48,064
	Yongin	Dwa	7	11.6	25.1	-3.1	1291.9	17.2	374.6	75.6	50.1	17.2	51.2	13,887
Gangwon	Hwacheon	Dwa	1	10.5	24.1	-5.5	1257.9	14.4	358.4	65.8	42.4	14.4	48.9	6,797
	Yeongwol	Dwa	11	11.2	24.3	-3.6	1205.1	19.1	294.8	73.5	49.4	19.1	50.3	11,135
	Jeongseon	Dwa	2	10.7	24.0	-4.0	1126.6	17.5	332.7	66.8	57.9	17.5	49.4	8,839
Chungbuk	Boeun	Cwa	14	11.2	24.3	-2.9	1305.3	23.4	306.0	82.2	56.6	23.4	50.4	28,898
	Chungju	Dwa	8	11.7	25.2	-3.2	1214.3	20.0	293.3	73.8	56.7	20.0	51.4	24,025
	Danyang	Dwa	2	11.7	25.0	-3.4	1049.9	14.9	253.2	61.6	48.8	14.9	51.3	2,688
	Jecheon	Dwa	1	10.3	23.8	-4.8	1359.3	20.3	356.0	85.1	57.4	20.3	48.6	15,838
Chungnam	Dangjin	Cwa	3	12.0	25.3	-2.0	1165.5	17.2	294.4	67.8	53.1	17.2	51.9	40,468
	Hongseong	Cwa	4	12.7	26.4	-1.1	1120.2	22.6	284.9	74.3	80.3	22.6	53.4	55,989
	Seosan	Cwa	1	12.1	25.3	-1.6	1253.9	23.5	283.5	74.7	54.1	23.5	52.2	33,009
	Cheonan	Cwa	4	12.0	25.2	-2.4	1218.7	19.0	299.7	67.5	55.5	19.0	52.0	34,062
Jeonbuk	Gimje	Cwa	2	13.3	26.6	-0.4	1089.9	23.1	246.8	69.9	52.4	23.1	54.7	43,513
	Namwon	Cwa	66	12.5	25.4	-1.3	1353.2	28.5	313.3	81.8	63.0	28.5	52.9	35,860

	Sunchang	Cwa	10	12.8	26.0	-1.5	1473.3	25.9	366.6	87.3	88.6	25.9	53.5	19,915
	Wanju	Cwa	5	13.0	26.2	-1.0	1296.1	25.7	310.4	86.5	50.9	25.7	54.0	30,871
	Imsil	Cwa	4	11.3	24.4	-2.5	1335.6	28.5	312.1	83.6	58.1	28.5	50.6	21,671
	Jangsu	Dwa	1	10.7	23.4	-3.0	1486.0	31.9	339.7	96.6	64.5	31.9	49.4	29,381
	Muju	Cwa	1	11.6	24.7	-2.6	1105.3	16.9	285.1	65.6	56.3	16.9	51.3	8,005
Jeonnam	Damyang	Cwa	1	13.2	25.8	0.0	1344.0	27.2	308.3	89.7	64.0	27.2	54.4	26,027
	Muan	Cfa	1	13.4	26.1	0.8	1290.1	29.6	271.3	96.5	72.3	29.6	54.8	33,215
	Gokseong	Cwa	10	13.0	25.8	-0.7	1332.9	25.1	321.9	79.7	63.8	25.1	54.0	16,707
	Gwangyang	Cwa	1	14.4	26.5	1.9	1424.3	20.8	308.2	108.9	68.6	20.8	56.8	3,253
	Hwasun	Cwa	12	13.4	26.0	0.1	1308.0	28.6	296.0	83.7	62.8	28.6	54.9	17,436
	Jangheung	Cwa	5	13.3	25.6	1.0	1471.8	28.2	316.5	108.5	78.7	28.2	54.6	47,975
	Suncheon	Cwa	8	14.8	26.6	2.5	1467.4	21.1	307.9	118.2	67.2	21.1	57.5	18,537
Gyeongbuk	Uljin	Cfa	1	12.8	23.9	1.4	1181.7	33.2	213.0	70.5	97.3	33.2	53.7	10,245
	Cheongdo	Cwa	2	12.9	25.7	-1.1	1196.9	20.1	255.4	85.7	57.0	20.1	53.8	24,800
	Goryeong	Cwa	1	12.8	25.6	-1.1	1235.4	18.0	283.4	83.9	65.0	18.0	53.7	16,467
	Sangju	Cwa	2	12.8	25.3	-0.9	1178.6	21.7	279.3	86.6	57.1	21.7	53.5	79,062
Gyeongnam	Geoje	Cfa	1	14.4	26.0	2.6	1930.3	37.0	367.6	177.2	112.2	37.0	56.8	2,212
	Goseong	Cwa	2	14.2	26.8	1.6	1470.4	25.6	304.0	133.2	84.5	25.6	56.4	19,793
	Changwon	Cwa	2	14.8	26.5	2.6	1534.1	27.7	304.5	133.8	78.5	27.7	57.5	11,954
	Sancheong	Cwa	2	13.0	25.1	0.4	1556.2	23.9	362.3	105.5	74.2	23.9	54.0	12,494
	Daegu	Cwa	4	14.5	26.7	1.1	1080.8	18.6	245.3	70.6	50.1	18.6	57.1	14,882

South Korean regions were classified at the city, county, and district levels. Case counts represent the number of confirmed AKAV cases between 1998 and 2020. Meteorological data were obtained from the Korea Meteorological Administration and represent 30-year means

(1991–2020). Cattle population between 2016 and 2020 is derived from Korea Institute for Animal Products Quality Evaluation (KAPE). Abbreviation. AKAV, Akabane virus; KG, Köppen–Geiger climate classification; Ta, annual mean temperature (°C); T_{max}, monthly mean temperature of the warmest month (°C); T_{min}, monthly mean temperature of the coldest month (°C); P_a, annual total precipitation (mm); P_{min}, precipitation of the driest month (mm); P_{smax} and P_{smin}, extreme value of monthly precipitation for summer half years (April to September, mm); P_{wmax} and P_{wmin}, extreme value of monthly precipitation for winter half years (October to March, mm); P_{th}, precipitation threshold.

Table S5. Regional Akabane virus cases and meteorological data in Japan.

Prefecture	KG zone	AKAV cases	T_a (°C)	T_{max} (°C)	T_{min} (°C)	P_a (mm)	P_{min} (mm)	P_{smax} (mm)	P_{smin} (mm)	P_{wmax} (mm)	P_{wmin} (mm)	P_{th}	Cattle Population (head)
Aichi	Cfa	24	16.2	28.2	4.8	1578.9	50.8	231.6	127.5	164.7	50.8	46.4	66,120
Chiba	Cfa	159	16.2	27.1	6.1	1454.7	56.8	204.7	110.4	225.7	56.8	46.4	68,980
Ehime	Cfa	91	16.8	28.1	6.2	1404.6	50.9	228.7	99.0	113.0	50.9	47.6	15,772
Fukui	Cfa	22	14.9	27.4	3.7	2270.8	137.2	239.8	137.2	304.0	153.8	43.8	3,522
Fukuoka	Cfa	153	17.3	28.4	6.9	1686.9	67.5	299.1	118.2	103.7	67.5	62.6	34,300
Fukushima	Cfa	23	13.4	25.5	1.9	1207.0	41.1	177.7	81.8	138.7	41.1	40.8	61,480
Gifu	Cfa	12	16.2	28.3	4.6	1860.7	65.9	270.9	162.4	161.6	65.9	46.4	37,626
Gunma	Cfa	13	15.0	26.8	3.7	1247.4	23.8	204.3	74.8	142.2	23.8	58.0	91,660
Hiroshima	Cfa	79	16.5	28.5	5.4	1572.2	46.2	279.8	131.4	118.3	46.2	61.0	32,606
Hokkaido	Dfa	20	9.2	22.3	-3.2	1146.1	54.6	142.2	54.6	114.5	77.6	32.4	1,313,780
Hyogo	Cfa	44	16.9	28.5	6.0	1277.8	38.4	187.9	100.6	118.0	38.4	47.7	66,200
Ibaraki	Cfa	73	14.1	25.6	3.3	1367.7	49.6	186.3	116.7	185.4	49.6	42.2	73,980
Ishikawa	Cfa	15	15.0	27.3	4.0	2401.5	138.0	233.4	138.0	301.1	157.2	44.0	6,442
Iwate	Cfa	119	10.6	23.5	-1.6	1279.9	48.0	197.5	85.4	108.7	48.0	35.2	132,700
Kagawa	Cfa	14	16.7	28.6	5.9	1150.1	39.4	167.4	74.6	120.1	39.4	47.4	24,778
Kagoshima	Cfa	85	18.8	28.8	8.7	2434.7	78.3	570.0	194.9	161.0	78.3	65.5	344,580
Kanagawa	Cfa	2	16.2	27.0	6.1	1730.8	64.7	241.5	139.0	240.4	64.7	46.4	10,504
Kochi	Cfa	6	17.3	27.9	6.7	2666.4	59.1	398.1	225.3	207.5	59.1	62.6	8,876
Kumamoto	Cfa	65	17.2	28.4	6.0	2007.0	57.2	448.5	144.9	124.8	57.2	62.4	170,500
Kyoto	Cfa	16	16.2	28.5	4.8	1522.9	53.3	223.6	117.0	143.2	53.3	46.4	9,826
Mie	Cfa	15	16.3	27.9	5.7	1612.9	47.2	276.6	129.0	186.1	47.2	46.6	34,778

Miyagi	Cfa	79	12.8	24.4	2.0	1276.7	33.9	192.6	90.2	150.6	33.9	39.6	99,860
Miyazaki	Cfa	96	17.8	27.8	7.8	2625.5	72.7	516.3	194.5	196.7	72.7	63.7	259,040
Nagano	Cfa	17	12.2	25.4	-0.4	965.1	44.4	137.7	56.9	100.3	44.4	38.4	36,840
Nagasaki	Cfa	29	17.4	28.1	7.2	1894.7	63.1	335.9	153.0	123.2	63.1	62.8	86,454
Nara	Cfa	20	15.2	27.3	4.2	1365.1	52.4	184.1	98.9	134.7	52.4	44.5	7,166
Niigata	Cfa	6	14.1	26.6	3.0	1845.9	94.4	222.3	94.4	225.9	112.0	42.2	18,990
Oita	Cfa	50	16.7	27.7	6.5	1727.0	47.1	313.6	119.7	144.8	47.1	61.4	60,860
Okayama	Cfa	296	16.3	28.5	5.1	1143.1	36.2	177.4	90.0	95.4	36.2	46.7	48,320
Okinawa	Cfa	24	23.3	29.1	17.3	2161.0	101.6	284.4	161.0	179.2	101.6	60.6	78,360
Osaka	Cfa	24	17.1	29.0	6.2	1338.3	47.0	185.1	101.9	136.0	47.0	48.2	1,978
Saga	Cfa	43	16.9	28.2	5.8	1610.0	85.8	219.0	117.3	140.7	85.8	61.8	54,238
Saitama	Cfa	6	15.4	27.1	4.3	1305.8	30.9	198.2	90.7	177.1	30.9	44.8	26,166
Shiga	Cfa	12	15.0	27.5	3.9	1610.0	85.8	219.0	117.3	140.7	85.8	44.0	21,760
Shimane	Cfa	109	15.2	27.1	4.6	1791.9	113.0	234.1	113.0	154.5	118.4	44.4	40,170
Shizuoka	Cfa	14	16.9	27.4	6.9	2327.3	79.6	296.6	186.5	250.3	79.6	47.8	33,640
Tochigi	Cfa	20	14.3	26.0	2.8	1524.7	37.5	217.2	121.5	174.4	37.5	56.6	133,020
Tokushima	Cfa	105	16.8	28.1	6.3	1619.9	41.9	271.2	104.3	199.5	41.9	47.6	27,058
Tokyo	Cfa	1	16.5	27.6	6.1	1598.2	56.5	224.9	133.7	234.8	56.5	47.0	2,142
Tottori	Cfa	23	15.2	27.3	4.2	1931.3	102.2	225.4	102.2	218.4	144.3	44.4	26,856
Wakayama	Cfa	16	16.9	28.4	6.2	1414.4	48.7	183.5	98.4	160.8	48.7	47.8	3,204
Yamagata	Cfa	39	12.1	25.0	-0.1	1206.7	63.0	187.2	63.9	105.1	63.0	38.2	50,000
Yamaguchi	Cfa	25	15.7	27.5	4.6	1927.7	70.5	342.6	168.1	145.6	70.5	59.5	17,638
Yamanashi	Cfa	7	15.1	27.1	3.1	1160.7	37.6	178.7	79.5	158.5	37.6	44.2	8,690

Japanese regions were grouped at the prefectural level. Case counts represent the number of confirmed AKAV cases between 1998 and 2020. Meteorological data were obtained from the Japan Meteorological Agency and represent 30-year means (1991–2020). Cattle population between 2016 and 2020 is derived from Portal Site of Official Statistics of Japan (e-STAT).

Abbreviation. AKAV, Akabane virus; KG, Köppen–Geiger climate classification; Ta, annual mean temperature (°C); T_{max}, monthly mean temperature of the warmest month (°C); T_{min}, monthly mean temperature of the coldest month (°C); P_a, annual total precipitation (mm); P_{min}, precipitation of the driest month (mm); P_{smax} and P_{smin}, extreme value of monthly precipitation for summer half years (April to September, mm); P_{wmax} and P_{wmin}, extreme value of monthly precipitation for winter half years (October to March, mm); P_{th}, precipitation threshold.

Table S6. Annual mean temperature and total precipitation data (1997-2020) and AKAV case numbers (1998-2020) of Okayama prefecture, Japan.

Years	AKAV cases	Annual mean temperature (℃)	Annual total precipitation (mm)
1997	-	16.1	1047.5
1998	77	17.3	1261
1999	82	16.5	957.5
2000	3	16.5	813
2001	0	16.3	1282.5
2002	3	16.6	827.5
2003	0	16.2	1190
2004	9	17.1	1480
2005	37	16.4	732.5
2006	0	16.4	1254
2007	0	17	773
2008	40	16.6	951.5
2009	23	16.6	1074
2010	1	16.7	1216
2011	21	16.3	1416.5
2012	0	16.1	1106
2013	0	16.4	1341
2014	0	16.1	1142.5
2015	0	16.2	1333.5
2016	0	16.6	1513

2017	0	15.7	1205.5
2018	0	16.3	1410
2019	0	16.5	921.5
2020	0	16.5	1154

AKAV case numbers of each year are compared with meteorological data for the same year (1998-2020) or prior year (1997-2019). AKAV case numbers of each year are compared with meteorological data for the same year (1998-2020) or prior year (1997-2019).
